# Provision of essential health package in public hospitals: a case of Homabay County hospitals, Kenya

**DOI:** 10.11604/pamj.2016.24.8.9280

**Published:** 2016-05-04

**Authors:** Shadrack Ochieng Opon

**Affiliations:** 1 Kenya Methodist University, Kenya

**Keywords:** Health Workers, drugs and supplies, Health infrastructure, patient satisfaction, organizational practices

## Abstract

**Introduction:**

Essential Health Packages (EHP) delivery is likely to strengthen service delivery. Healthcare utilization rate is 77% for the sick. 44% and 18% who don't seek care are hindered by cost and distance respectively. The overall child mortality rate in Kenya is 121/1000. In Homabay County, child mortality rate is 91/1000, and maternal mortality rate of 583/100000. The study looked into the provision of EHP in public hospitals in Homabay County.

**Methods:**

Cross-sectional research design was used. Two hospitals were conveniently due to their municipality location. The study targeted 213 Health workers and 350 patients. Stratified sampling and proportionate sampling was used among different health workers. Sample size was determined by Yamane Formula. The study sampled 138 health workers and 186 patients. Questionnaire and key interview guide were used to collect data.

**Results:**

There are inadequate health workers based on 138 (100%) health workers. Insufficient drugs were reported by 138 (100%) health workers, and 120 (64.5%) patients. 115 (83.3%) health workers say ambulances are not operational. 26 (18.8%) health workers noted lack medical equipment, 138 (100%) are aware of patients referred elsewhere due to lack of medical equipment. 153 (82.3%) and 135 (72.6%) patients’ health access is hindered by cost and distance respectively. 159 (85.5%) patients don't always find services needed. 159 (85.5%) patients affected by long waiting time.

**Conclusion:**

Low service provision/utilization rate in Homabay County results from lack of health workers, inadequate drugs, poor health infrastructure, and lack of access in terms of affordability, availability and distance.

## Introduction

**Figure 1:**
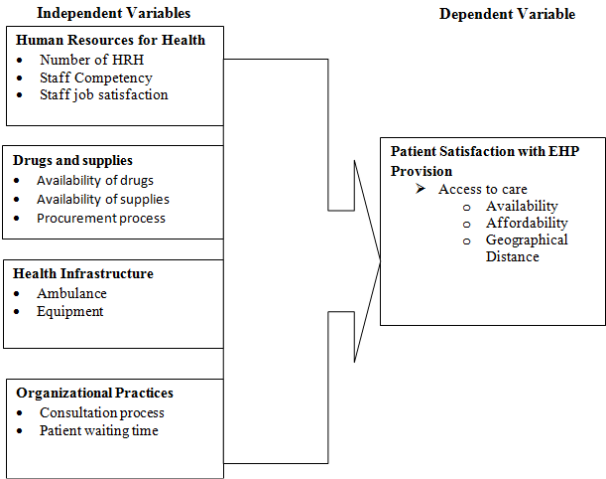
Conceptual framework

Availability of health care services does not guarantee that they will be optimally used by patients. Financial or geographical access remains a barrier to health services. In Kenya, the poorer masses, those living below the national poverty line, constitute approximately 52% of the population [1]. 82% of lost healthy life-years were attributed to communicable diseases [2]. The overall under-five child mortality rate is approximately 121 per 1000 live births, or roughly doubles the global average. However, this number drops significantly, to 91 per 1000, for the wealthiest 20% of the population, while it jumps to nearly 150 for the poorest 20% [3]. According to recent data, the health care utilization rate in Kenya is approximately 77% for those who are sick, meaning that a large percentage of the population does not seek care despite being ill [4]. Among those Kenyans who are ill and do not choose to seek care, 44% were hindered by cost. Another 18% were hindered by the long distance to the nearest health facility [4]. In Homabay County, lack of access to health services is among the top reasons for low service delivery and utilization. According to the survey conducted by UNICEF in January 2014 in Homabay County, the County has one of the highest under-five mortality rates in Kenya (at 91/1000 live births) [5]. With the poverty rate of Homabay County at 44% compared to the national average of 47%, people of Homabay cannot consistently seek health care without risking financial catastrophe. Homabay has unacceptably high maternal mortality rate, estimated at 583 per 100000 live births, compared to the national average of 488 per 100000 live births [5]. Therefore, addressing factors influencing the provision of essential health package in Homabay can be a solution to strengthen service delivery and improve access to health care. The study aims were: to determine how human resources for health influence the provision of Essential Health Package in Homabay County; to establish how availability of drugs influence the provision of Essential Health Package in Homabay County; to identify the role of health infrastructure in the provision of Essential Health Package in Homabay County; to evaluate the relevance of organizational practices in the provision of Essential Health Package in Homabay County. This study strengthens service delivery pillar. It provides recommendations on how to increase access to Essential Health Package, delivery and utilization of the same, in order to enhance utilization and affordability of healthcare especially among the poor and vulnerable population. The study looks into the competency of health workforce, health infrastructure, the availability of drugs and supplies which are key factors in successfully delivering the essential health package as in Figure 1.

## Methods

This was a cross-sectional research design with mixed method approach conducted in the two hospitals to answer the research questions. The study was conducted in public hospitals in Homabay County. The focus of the study was on the two major tiers 3 and tier 2 hospitals (Homabay County Hospital and Mbita Sub-County Hospital respectively) because of their location within the municipality. In addition, these two hospitals are the largest facilities serving the largest number of the county's population, which is the reason the study focuses on them. The study targeted 213 health workers, out of which 138 (Pharmacists 5, clinicians 13, HMIS Officers 8, Nurses 104, and Hospital Managers 8) was sampled, and 350 patients out of which 186 was sampled. The 8 hospital managers were the key informants The study used Yamane's Simplified Sample Formula for proportions which is suitable for small population and reducing the sample size slightly. n=N/ (1+N (e^2^)) Where: n is the sample size; N is the population size; e is the level of precision (margin of error)

For Healthcare Workers: n1=213/ (1+213(0.05)^2^); n1=138.989. Thus, n1=138 Distribution of health workers by cadre is shown in Table 1

**Table 1:** Distribution of healthcare workers sampled by cadres

	Homabay County Hospital	Sampled	Mbita Sub-County Hospital	Sampled	Total N (%)	Total sampled
Pharmacists	5	3	3	2	8 (3.76)	5
Clinicians	13	8	7	5	20(9.39)	13
HMIS Officers	9	6	4	2	13(6.10)	8
Nurses	113	73	47	31	160(75.11)	104
Hospital Managers	8	5	4	3	12(5.63)	8
Total Population	148	95	65	43	213(100)	*n*_*1*_=138

For the Patients: n2=350/ (1+350(0.05)^2^) n2=186.666. Thus, n2=186. Sampling of patients is shown inTable 2

**Table 2:** Sampling of patients

	Homabay County Hospital	Mbita sub-County Hospital	Total	Sample Size
Average Daily Patients	270	80	350	
Sampled	143	43	186	*n*_2_=186

Stratified sampling method was used for the staff based on cadres in order to have a sample population that is representative of all the cadres. Proportionate sampling method was then be used to divide the sample size proportionately by percentage of the total population. That is, the total population per cadre over the general population multiplied by the determined sample size. This gave the sample size per cadre to be used in the study. Proportionate sampling method ensured that the cadres are represented proportionately in the final determined sample size as illustrated in Table 1 and Table 2. Both stratified sampling and proportionate sampling was used among the patients to reach a sample size for each facility. The study used daily attendance rate to ensure a manageable sample size because monthly or annual attendance rate would have given a huge sample that may not have been achieved during the study as data collection was carried out in a day. An exit interview was then conducted to gather information concerning service utilization. Sample population included only those who are directly concerned with the delivery of Essential Health Packages. These included all registered employees in the hospitals. Patients (both inpatients and outpatients) who were in the hospital at the time of the interview were sampled randomly at the exit point. Student staff and those who were absent at the time of the study were excluded. Patients in the surgical department were excluded from the study because of their delicate health condition. It would have not been easy for them to respond to the questionnaire appropriately. This study used a structured questionnaire and a key informant guide. There was a pretest of the research instruments conducted in Homabay County Hospital as a means to preview the likelihood of successful study. Based on the result of the pretest study, the questionnaire was redrafted to ensure the right data was collected in the actual study. The accuracy was tested during the pre-test among the respondents sampled randomly in the hospital to ensure validity. While some questions yielded the expected results, some questions were restructured, rewritten based on the way the respondents understood them, in order to get accurate responses during the actual study. The reliability of the questionnaires was measured by giving the same questionnaires to the same respondents on two separate occasions. A reliability coefficient of 0.8 was set to ensure good reliability. The responses on two separate occasions were correlated, and a reliability coefficient of 0.9 was obtained. The study employed both quantitative and qualitative methods of study during data collection, particularly structured questionnaire and interview guide. A structured questionnaire, self-administered was used among the target population (pharmacists, clinicians, HMIS officers, and nurses) to collect data. An exit interview was carried out among patients at the exit point of the facilities to gather information on satisfaction with service provision. Key Informant Interview (KII) was used among the hospital departmental heads to gather information on hospital infrastructure and staff competency levels. Quantitative data was coded and analysis done with the use of SPSS. Regression was used to analyze qualitative data. This involved both the descriptive and inferential statistics. Descriptive statistics was used to describe the status of both the independent (HRH, availability of drugs and supplies, health infrastructure, and organizational practices) and dependent variable (Patient satisfaction with the services). Inferential statistics was used to generate conclusions on the influence of independent variables on the dependent variable. Presentation of data was done with the use of tables. Prior to the commencement of the study, permission was obtained from respective authorities of the hospitals and Kenya Methodist University. Informed consent was also requested from all the respondents.

## Results

### The influence of the human resources for health

The presence of health workers in Homabay County is inadequate. Based on the findings and discussion above, with 138 (100%) of health workers admitting that there is a need for more health care workers, and majority, 138 (100%) saying they are not satisfied with the existing number of health workers, it is evident that there are not enough health care workers in Homabay County hospitals. This infers that there is minimal service provision in the hospitals because of lack of enough health care workers. The education level of the most existing health care workers, 100 (72.5%) are up to diploma level, besides, a huge number of health workers 109 (79%) and 97 (70.3%) are not satisfied with their income and job respectively. The regression result shows that patient satisfaction with the services is directly influenced by the human resources for health. A positive change in any of the human resources for health variables (number of HRH, level of education, job satisfaction) will lead to a positive change in the patient satisfaction with services. This is indicated by the regression results in Table 3.

**Table 3:** Relationship between human resources for health and patient satisfaction with services

Coefficients^a^											
Model		Unstandardized Coefficients		Standardized Coefficients	t	Sig.	95.0% Confidence Interval for B		Correlations		
		B	Std. Error	Beta			Lower Bound	Upper Bound	Zero-order	Partial	Part
1	(Constant)	1.35	0.33		4.09	0.00	0.69	1.20			
	Staff Competency	0.10	0.061	0.14	1.66	0.10	0.22	0.02	0.11	0.14	0.14
	Job satisfaction	0.11	0.07	0.14	1.68	0.09	0.25	0.02	0.11	0.14	0.14
	Number of health workers	0.25	0.09	0.23	2.70	0.01	0.07	0.43	0.18	0.23	0.22

a Dependent Variable: patient satisfaction with service provision

**Model Statement**

Y = a+bX1+cX2+dX3

Y = Patient Satisfaction with Services

X1 = Staff Competency

X2 = Job satisfaction

X3 = Number of health workers

### The effect of drugs and supplies in the provision of EHP

Availability of drugs and supplies in Homabay County is wanting. 100% (138) of the health workers said they do not have enough drugs in the hospitals. 120(64.5%) patients have also rated drug availability as poor. It is evident that there are not enough drugs in the hospitals. Majority, 70(50.7%) of the health workers have asked the patients to buy drugs from elsewhere, making service delivery a challenge. While the hospitals may have an existing procurement system, it is evident that the drugs and supplies are received over three months later after making an order as indicated by 97(70.3%) of health workers. Despite the delay in the receipt of the drugs, 138(100%) say that not all the drugs and supplies ordered reach the facilities. In addition, the drugs that get to the hospital sometimes expire without use. This is an indication that a lot of drugs ordered are not in demand, based on the fact that 180(96.8%) patients are sometimes asked to buy drugs elsewhere, which is a deterrent to seeking health care, out of which 34(18.6%) and 18(9.8%) do not end up buying these drugs. Patient satisfaction with the services is directly influenced by the availability of drugs and supplies. A positive change in any of the variables of the drugs and supplies (availability of drugs, availability of supplies, procurement procedure) will lead to a positive change in the patient satisfaction with services. This is indicated by the regression results in table 4.

**Table 4:** Relationship between drugs and supplies and patient satisfaction with services

Coefficients^a^											
Model		Unstandardized Coefficients		Standardized Coefficients	t	Sig.	95.0% Confidence Interval for B		Correlations		
		B	Std. Error	Beta			Lower Bound	Upper Bound	Zero-order	Partial	Part
1	(Constant)	0.86	0.34		2.49	0.01	0.18	1.53			
	Satisfaction with Procurement Process	0.03	0.05	0.05	0.59	0.56	0.12	0.06	0.05	0.05	0.05
	Availability of Drugs	0.14	0.06	0.20	2.32	0.02	0.02	0.25	0.19	0.20	0.20
	Availability of Supplies	0.11	0.17	0.06	0.67	0.51	0.44	0.22	0.02	0.06	0.06

a Dependent Variable: patient satisfaction with services

**Model Statement**

Y = a+bX1+cX2+dX3

Y = Patient satisfaction with services

X1 = Satisfaction with Procurement Process

X2 = Availability of Drugs

X3 = Availability of Supplies

### Role of health infrastructure in the provision of EHP

Health infrastructure contributes significantly in the provision of services in Homabay County. While 134 (97.1%) of the health workers said the hospital have ambulances, 115 (83.3%) of the health workers said the ambulances are not operational. In addition, 112 (81.2%) of the health workers admit that they have the basic medical equipment required to offer service, however, 138 (100%) of the health workers have referred or aware of a patient referred elsewhere due to lack of basic equipment required for the service. Health infrastructure directly influences the patient satisfaction with the services. A positive change in any of the variables of the health infrastructure (ambulances, medical equipment) will lead to a positive change in the patient satisfaction with services. This is indicated by the regression results in table 5.

**Table 5:** Relationship between health infrastructure and patient satisfaction with services

Coefficients^a^											
Model		Unstandardized Coefficients		Standardized Coefficients	t	Sig.	95.0% Confidence Interval for B		Correlations		
		B	Std. Error	Beta			Lower Bound	Upper Bound	Zero-order	Partial	Part
1	(Constant)	0.90	0.13		6.71	0.00	0.63	1.16			
	Availability of Operational Ambulances	0.12	0.19	0.06	0.63	0.53	0.26	0.50	0.08	0.05	0.05
	Medical Equipment Availability	0.18	0.09	0.20	2.00	0.05	0.36	0.01	0.20	0.17	0.17
	Existing Medical Engineering Department	0.02	0.07	0.02	0.22	0.83	0.15	0.12	0.05	0.02	0.02

a Dependent Variable: Patient Satisfaction with Services

**Model Statement**

Y = a+bX1+cX2+dX3

Y = Patient Satisfaction with Services

X1 = Availability of Operational Ambulances

X2 = Medical Equipment Availability

X3 = Availability of Medical Engineering Department

### Importance of organizational practices in the provision of EHP

There are poor organizational practices in the Homabay County hospitals. Based on the results of the study facilities have labeled their service rooms. However, the arrangement is poor and confusing to the patients based on the responses of 121(65.1%) patients. This infers that patients take a lot of time to locate the rooms, besides the up to 60 minutes waiting time cited by 97(52.2%) patients. Some service points such as Laboratory have the maximum waiting time before service can be offered based on the responses of 67.4% (93) health workers. This might discourage the patients from seeking health care. In addition, 159 (85.5%) patients have failed to seek health care because of long waiting time. Organizational practices directly influence the patient satisfaction with the services. A positive change in any of the organizational practices variables (Consultation rooms, patient waiting time) will lead to a positive change in the patient satisfaction with services. This is indicated by the regression results in table 6.

**Table 6:** Relationship between organizational practices and patient satisfaction with services

Coefficients^a^											
Model		Unstandardized Coefficients		Standardized Coefficients	t	Sig.	95.0% Confidence Interval for B		Correlations		
		B	Std. Error	Beta			Lower Bound	Upper Bound	Zero-order	Partial	Part
1	(Constant)	0.30	0.15		1.97	0.05	0.01	0.60			
	Number of Consultation Rooms	0.11	0.04	0.25	2.76	0.01	0.03	0.19	0.34	0.23	0.22
	Satisfaction with the waiting time	0.20	0.07	0.26	2.92	0.01	0.07	0.34	0.36	0.24	0.23
	Satisfaction with flow of services	0.07	0.05	0.13	1.54	0.13	0.02	0.16	0.01	0.13	0.12

a Dependent Variable: patient satisfaction with services

**Model Statement**

Y=a+bX1+cX2+dX3

Y= Patient Satisfaction with Services

X1= Number of Consultation Rooms

X2= Satisfaction with the waiting time

X3= Satisfaction with the flow of services

### Patient satisfaction with EHP provision

Patient satisfaction with the services greatly influences service utilization in Homabay County. Based on the study, 149(80.1%) patients do not find services affordable. In addition, 153 (82.3%) of the patients have failed to seek health care because of cost. Nevertheless, 108(58.1%) patients take between 2 and 4 hours to get to the hospitals. 135(72.6%) patients said they have failed to seek health care because of the distance. 159 (85.5%) patients do not get all the services when they need them. In addition, 143(76.9%) have been referred elsewhere because the service they needed was not available. The study has documented that patient satisfaction with the service provision is directly linked to access to the services based on the cost, availability, and geographical distance.

## Discussion

### The influence of the human resources for health

The findings are similar to those in [6] that lack of healthcare professionals can inhibit access to services by limiting the supply of available services. On the part of the patient satisfaction with the service provision, it is evident that the lack of enough health workers is responsible for low service provision, which means patients are not satisfied with the services resulting from inadequate health workers. Therefore, availability of human resources for health directly influences satisfaction of patients with the services. This is justified by the positive regression coefficient of 0.25 of number of health workers, indicating that an increase in the number of health workers by one health worker will result to an increase in the patient satisfaction with services in terms of availability of services by 0.25 units provided all other factors are held constant as shown in Table 3. The results indicate minimum qualifications with minimal internal and external training of health workers. In addition, the regression results shows that a positive change in the level of education of health workers by one unit will result to an increase in patient satisfaction with services by 0.10 units provided all factors are held constant as indicated in Table 3. This study agrees with those in [7] that lack of highly qualified health workers is a direct impediment to the delivery of health services. Many health care seekers are forced reschedule medical visits of seek medication elsewhere due to lack of qualified professionals [7]. Therefore, the lack of enough highly qualified personnel infers dissatisfaction with the service provision in terms of availability of service among the patients as many are asked to seek services elsewhere, or reschedule a visit. This is a direct indication of low satisfaction with work, and as shown in Table 3, a positive change in Job satisfaction by one unit will result to an increase in the patient satisfaction in terms of availability of services by 0.11 units provided other factors are held constant. These findings are justified by [7], who found out that the signs of job dissatisfaction result in high absenteeism, low productivity, labor unrest, high labor turnover, and industrial action, leading to low number of people seeking health care due to fear of poor quality services [7]. Therefore, patients receive poor services resulting from the dissatisfied personnel. This ultimately leads to patient dissatisfaction with the services rendered poorly without concern due to fatigue.

### The effect of drugs and supplies in the provision of EHP

The study found out that the hospitals do not have all the drugs they prescribed to the patients as well as the supplies they require to offer most services. The study shows that availability of drugs is influences patient satisfaction with the service based on Table 4, which shows that a positive change in Availability of Drugs by one unit will result to an increase in the patient satisfaction with availability of services by 0.14 units provided other factors are held constant. These findings show a similar trend as those found by Ensor et al. (2009) in Uganda, which indicated that 60% of the people who required medical attention were turned away from the pharmacy due to lack of drugs prescribed by the clinicians [8]. In addition, unreliability of obtaining drugs and medical supplies compromises the timely provision of quality services. This infers that patients are not satisfied with the service provision as majority is asked to buy drugs elsewhere. These results are consistent with assertion in [9] that it is necessary that hospital managers have an effective procurement system with a pre-set re-order level that is consistent with the needs of the people, and delivers drugs as ordered in time. In addition, [9] concluded that untimely deliver of drugs and supplies continue to be one of the causes of drug unavailability in the hospitals leading to prevalence of diseases which would have otherwise been treated.

### Role of health infrastructure in the provision of EHP

The results are an indication of lack of emergency medical responses that requires ambulances. This is in agreement with [3] which stated that only handful facilities have operational ambulances leading to poor medical responses in case of emergencies. As seen in Table 5 the regression results show that a positive change in availability of Operational Ambulances by one unit will result to an increase in patient satisfaction with services by 0.12 units provided all factors are held constant. Therefore, lack of operational ambulances influences satisfaction of patients with the services because people who are referred elsewhere to seek services they would have received may not even come back to the facility in a later date. The regression results in Table 5 show that a positive change in Availability of Medical Equipment by one unit will result to an increase in the patient satisfaction with services by 0.18 units provided other factors are held constant. Therefore, availability of medical equipment directly influences availability of service, hence, patient satisfaction. It is, hence evident that the facilities lack maintenance systems for the existing medical infrastructure leading to lack of the basic services that require medical equipment. When a patient is referred elsewhere for treatment due to lack of the required equipment, or when health workers are not able to respond to emergency cases due to lack of operational ambulances, the public is denied services which explains the role of health infrastructure in the delivery of services. The findings are consistent with those of [7], who deduced that facilities require an effective maintenance system to keep running. A lot of funds are channeled in buying new equipment while others that could be fixed are thrown away [7]. It is important for hospitals to have all their machines running in order to provide the services that require them to the people. The lack of functional medical engineering department influences satisfaction of patients with the services as showed by the regression result in Table 5 that an increase in the availability of Medical Engineering Department by one unit will result to an increase in patient satisfaction with services by 0.02 units.

### Importance of organizational practices in the provision of EHP

Based on the regression result in Table 6, a positive change in the number of Consultation Rooms by one unit will result to an increase in patient satisfaction with services by 0.112 units provided all factors are held constant. This is in agreement with [10] who found out that 30% of patients avoided facilities with poorly arranged consultation rooms. These patients cited that they do not like the manner in which they have to move from one room to another in order to get help. However, when there are many consultation rooms offering the same services, then many patients are likely to be served. The stages through which patients undergo to access health services need to be reduced in such a way that patients can access health services at one point, and go back home [10]. The more complicated the procedure for receiving service is, the less the satisfaction of patients with the service. Therefore, consultation process is a major determinant of whether or not the patients will seek the services, as revealed by the regression result in Table 6 that an increase in the Satisfaction with the flow of services by one unit will result to an increase in the patient satisfaction with services by 0.07 units. Indeed, a positive change in Satisfaction with Patient Waiting Time by one unit will result to an increase in the patient satisfaction with services by 0.20 units provided other factors are held constant as shown in Table 6. These findings are in agreement with those of [11], which documented that long waiting time reduces patient satisfaction, and discourages patients from seeking care. Therefore, health managers should make efforts to reduce the waiting time. As seen in the study, while services may be available, waiting time can be a deterrent to delivering and utilizing the services.

### Patient satisfaction with EHP provision

153 (82.3%) of the patients have failed to seek health care because of cost. These findings are in agreement with [4] which documented that 44% of Kenyans who are ill do not seek health care because of the high cost [4]. When the services are not affordable, patients are become less satisfied, and fail to seek services. 135 (72.6%) of the patients said they have failed to seek health care because of the distance. The study is justified by [12] who found out that distance from the facility determines whether people seek health services or not, as some people forgoes medical appointments due to fear of long distances. When the distance to the nearest facility is too long, there is the less satisfaction with the services because patients do not seek the services at all when the facilities are far away. 159(85.5%) of the patients do not get all the services they require. Based on the study by [12], availability of services is an important determinant of whether or not patients will seek services. The study found out that about 54% of people who are ill do not seek health care because of lack of services in the facilities (Bobadilla, 2008). Patients are extremely dissatisfied with the services when they make all the attempts to get the services but are asked to seek them elsewhere.

## Conclusion

Based on the findings and discussions above, the study found out that the provision of EHPs are directly influenced by human resources for health, availability of drugs, health infrastructure, and organizational practices. This is justified by the correlations and regression computed in the respective sections. In addition, the study has documented that patient satisfaction with the service provision is directly linked to access to the services based on the cost, availability, and geographical distance. Therefore, utilization of service would be increased when satisfaction level is elevated. Similarly, patient satisfaction is a product of adequate drugs and supplies, competent human resources for health, availability of health infrastructure, and convenient organizational practices. This is evident in the study's conceptual framework. In general, the following conclusions can be generalized: the low service provision in Homabay County can be attributed to the existing challenges in human resources for health; the provision and utilization of services is low in Homabay County because of inadequate drugs and supplies; the provision and utilization of services is low in Homabay County because of the lack of health infrastructure; low service provision and utilization in Homabay County can be attributed to poor organizational practices; the satisfaction level of the patients with the services in Homabay County is low, translating to low service utilization because of the cost of services, unavailability of basic services, and geographical distance.

### What is known about this topic

- There is high under-five (91 per 1000 live births) and maternal mortality (583 per 100000) rate in Homabay County;
- Lack of access to health services is responsible for low utilization of health services in Homabay County.

### What this study adds

- Inadequate drugs and supplies and poor health infrastructure are responsible for the high mortality rates in Homabay County;
- Cost of health services, geographical distance to the nearest facility, and patient waiting time impairs utilization of health services in Homabay County;
- Lack of enough human resources for health is a deterrent to the provision of health services in Homabay County.
